# Antileishmanial and Cytotoxic Compounds from *Valeriana wallichii* and Identification of a Novel Nepetolactone Derivative †

**DOI:** 10.3390/molecules20045740

**Published:** 2015-04-01

**Authors:** Jan Glaser, Martina Schultheis, Heidrun Moll, Banasri Hazra, Ulrike Holzgrabe

**Affiliations:** 1 Institute of Pharmacy and Food Chemistry, University of Wuerzburg, Am Hubland, 97074 Wuerzburg, Germany; E-Mail: jan.glaser@uni-wuerzburg.de; 2 Institute for Molecular Infection Biology, University of Wuerzburg, Josef-Schneider-Str. 2, 97080 Wuerzburg, Germany; E-Mails: martina.schultheis@uni-wuerzburg.de (M.S.); heidrun.moll@uni-wuerzburg.de (H.M.); 3 Department of Pharmaceutical Technology, Jadavpur University, Kolkata-700032, India; E-Mail: banasrihazra@yahoo.co.in

**Keywords:** *V. wallichii*, antileishmanial, cytotoxicity, podophyllotoxin, valtrates, novel nepetolactone derivative

## Abstract

The chloroform extract of *Valeriana wallichii* (*V. wallichii*) rhizomes was investigated to elucidate the structures responsible for reported antileishmanial activity. Besides bornyl caffeate (**1**, already been reported by us previously), bioassay-guided fractionation resulted in two additional cinnamic acid derivatives **2**–**3** with moderate leishmanicidal activity. The structure of a novel nepetolactone derivative **4** having a cinnamic acid moiety was elucidated by means of spectral analysis. To the best of our knowledge villoside aglycone (**5**) was isolated from this plant for the first time. The bioassay-guided fractionation yielded two new (compounds **6**–**7**) and two known valtrates (compounds **8**–**9**) with leishmanicidal potential against *Leishmania major* (*L. major*) promastigotes. In addition, β-bisabolol (**10**), α-kessyl alcohol (**11**), valeranone (**12**), bornyl isovalerate (**13**) and linarin-2-*O*-methylbutyrate (**14**) were identified. This is the first report on the isolation of 4'-demethylpodophyllotoxin (**15**), podophyllotoxin (**16**) and pinoresinol (**17**) in *V. wallichii*. In total thirteen known and four new compounds were identified from the extract and their cytotoxic and antileishmanial properties were evaluated.

## 1. Introduction

**Figure 1:**
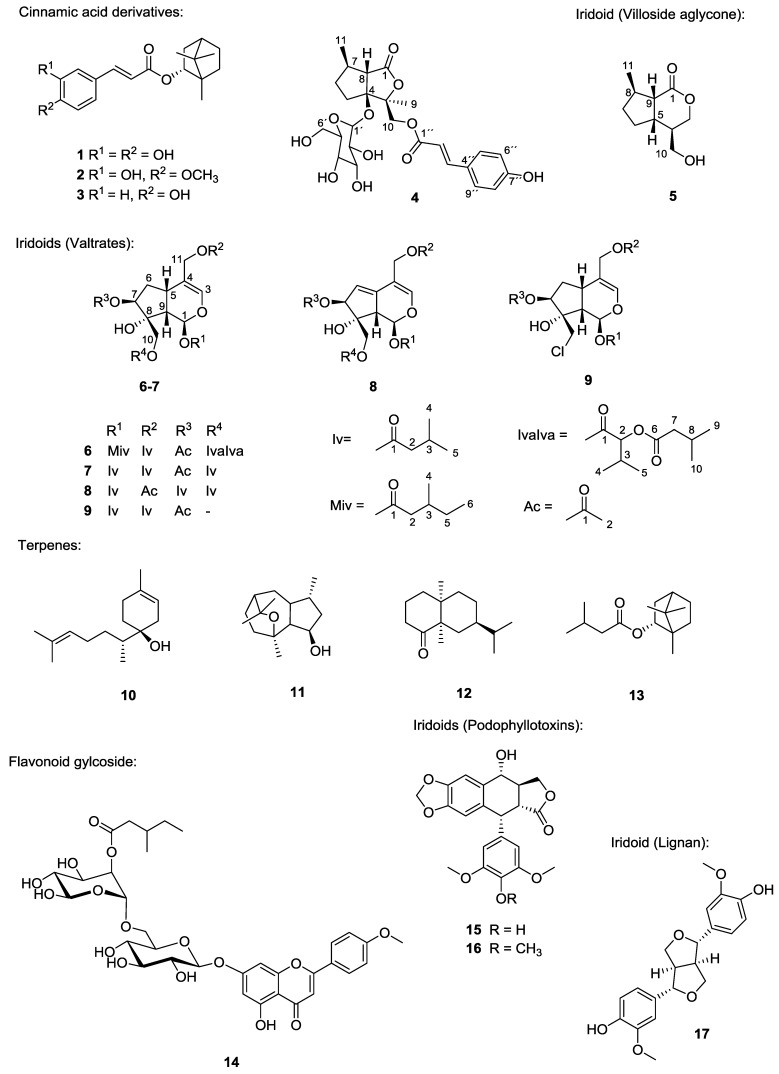
Isolated and identified compounds from *V. wallichii* extract. Abbr.: Iv = isovaleryl; Miv = β-methylisovaleryl; IvaIva = α-isovaleroyloxyisovaleryl; Ac = acetyl.

*Valeriana wallichii* DC (syn. *V. jatamansi* Jones) is endemic to the Himalayan regions of India, Nepal and China and is used in folk and ayurvedic medicine, for instance, against sleep disorders, skin disorders or pain [1]. Antimicrobial [2], anti-inflammatory [3], insecticidal [4], antiviral [5] and antioxidant [6,7] properties of the extract are known. Additionally, the antileishmanial activity of the chloroform extract of the rhizomes was reported previously [8] and studies to isolate and identify the responsible compounds by bioassay-guided fractionation were undertaken and produced bornyl caffeate (**1**) [9]. In continuation of our search for antileishmanial compounds in *V*. *wallichii* we herein reveal the antileishmanial activity of valtrates and we isolated additional cytotoxic compounds which are to date unheard in *Valeriana*. We successfully isolated and characterized two more cinnamic acid derivatives **2**, **3** showing leishmanicidal properties. Additionally, we discovered a nepetolactone derivative **4** and villoside aglycone **5**, both being novel natural products. Two new (compounds **6**–**7**) and two known valtrates (compounds **8**–**9**) were found. For all valtrates we could show strong antileishmanial activity. Furthermore, β-bisabolol (**10**), α-kessyl alcohol (**11**), valeranone (**12**), bornyval (**13**), linarin-*O*-2-methylbutyrate (**14**), 4'-demethylpodophyllotoxin (**15**), podophyllotoxin (**16**) and pinoresinol (**17**) were isolated from the extract (*cf.* Figure 1).

## 2. Results and Discussion

### 2.1. Bioassay-Guided Fractionation of the Extract

**Figure 2:**
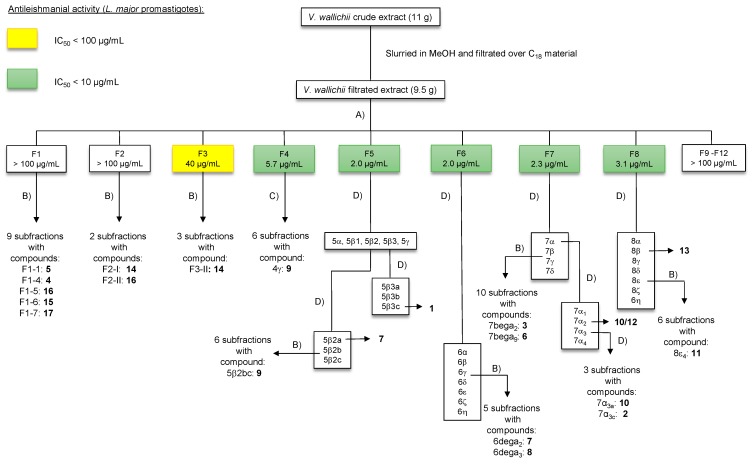
Overview of the bioassay-guided fractionation of *V. wallichii* extract. *Method A*: preparative HPLC (250 mm × 10 mm, 5 µm, Nucleosil 100-5); H_2_O (a): MeOH (b), gradient: 70% b (0 min), 75% b (7 min), 100% b (25 min), 70% b (30 min); flow rate: 3.3 mL/min. *Method B*: preparative HPLC (125 mm × 10 mm, 5 µm, Nucleodur Sphinx RP); H_2_O (a): CH_3_CN (b), gradient: 10% b (0–2 min), 30% b (2–15 min), 65% b (15–22 min), 10% b (22–24 min); flow rate: 4.4 mL/min. *Method C*: flash chromatography (silica gel; MeOH/CHCl_3_, 4.8:0.2, *v/v*). *Method D*: column chromatography (silica gel; MeOH/CHCl_3_, 4.8:02, *v/v*).

The dried chloroform extract of *V. wallichii* rhizomes was slurried in MeOH, filtered and fractionated into 12 fractions F1–F12 by preparative HPLC. After screening for antileishmanial activity on *L. major* promastigotes *in vitro* the most promising fractions F4–F8 were further investigated by subjecting them to preparative HPLC and multiple column chromatographic separations (*cf.* Figure 2) which led to the isolation and identification of highly active valtrates **6**–**9** and cinnamic acid derivatives **1**–**3** with moderate antileishmanial activity. β-Bisabolol (**10**) and valeranone (**12**) were found in a subfraction of F7 and bornyl isovalerate (**13**) and α-kessyl alcohol (**11**) were isolated from fraction F8. The non-active fractions F1–F3 gave the highest amount of substances. To avoid missing small amounts of potentially active compounds we have carefully investigated these fractions. This resulted in the discovery of a novel cinnamic acid derivative **4** with a nepetolactone skeleton and villoside aglycone **5**. From these fractions 4'-demethylpodophyllotoxin (**15**), podophyllotoxin (**16**), pinoresinol (**17**) and linarin-*O*-2-methylbutyrate (**14**) were isolated as well (*cf.* Figure 1).

### 2.2. Structure Elucidation of Novel Nepetolactone Derivative ***4***

**Figure 3:**
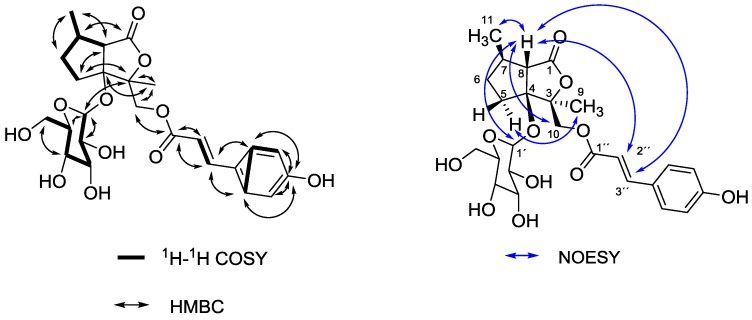
Key ^1^H-^1^H-COSY, HMBC and NOESY correlations of **4**.

Compound **4** was isolated as a yellowish syrup after multiple fractionation of F1. The IR spectrum showed absorption bands for hydroxyl groups (3382 cm^−1^), carbonyl functions (1749 and 1697 cm^−1^) and a double bond (1603 cm^−1^). The ESI-MS investigation yielded mass signals at *m/z* 506.9 [M−H]^−^, 621.4 [M−H+TFA]^−^ and 326.8 [M−Glc]^−^ in the negative mode, respectively, supporting the proposed structure. In the positive mode multiple adducts (526.2 [M+H_3_O]^+^, 531.1 [M+Na]^+^) were detected besides the molecular ion signal at *m/z* 509.0 [M+H]^+^. Fragmentation of *m/z* 526.2 and 531.1 gave a fragment of *m/z* 347.1 [M−Glc+Na]^+^. In the NMR spectra the characteristic ^1^H and ^13^C signals for a *trans*-cinnamic acid with a *para*-substituted phenyl ring were present. ^13^C signals in the range of 70–80 ppm represented a sugar moiety. The assignment of the sugar as β-d-glucose was supported by comparison of the coupling constants of the protons to data from the literature [10] while the connectivity of the sugar and the cinnamic acid derivative to the nepetolactone skeleton [11] was established by HMBC cross peaks. Interpretation of coupling constants and NOESY experiments revealed the stereochemistry (*cf.* Figure 3): NOESY correlations of H-8 with other protons were the most prominent ones. H-8 gave NOESY cross peaks with H-1' of the sugar moiety, H-10, H-2" and H-3" of the cinnamic acid rest and the H-11 protons of the methyl group which led to the conclusion that all these groups are in close spatial proximity. The cinnamic acid seems to be located in the space above H-8. Therefore the proposed configuration is consistent with the configuration of the villoside aglycone **5** and other similar compounds from *Valeriana* [12]. Additional evidence came from a weak NOESY correlation between the protons of the C-9 methyl group and H-5_b_. Thus, compound **4** was assigned to be {[(3*S*,4*S*,7*R*,8*S*)-1-oxo-3,7-dimethyl-4-(*O*-β glucopyranosyl)oxy]hexahydrocyclopenta-[*c*]furan-3-yl}-methyl-*trans*-4-hydroxycinnamate.

### 2.3. Structure Elucidation of Villoside Aglycone ***5***

**Figure 4:**
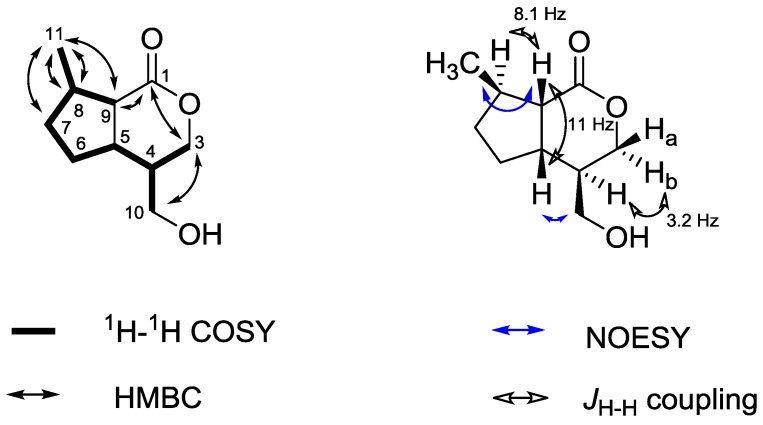
Key ^1^H-^1^H-COSY, HMBC and NOESY correlations of villoside aglycone **5**.

Villoside is an iridoid glycoside first isolated by Taguchi *et al*. [13] from *Patrinia villosa* Juss., a *Valerianaceae*. However, the villoside aglycone was only obtained semi-synthetically [13]. This is the first report on the isolation of the aglycone **5** directly from a plant, after chromatographic separation of F1 (*cf.* Figure 2). Similar compounds (jatamanines) have been isolated from *V. jatamansi* before [12]. Compound **5** was obtained as a yellow syrup. The IR spectrum is characterized by absorption bands at 3387 cm^−1^ indicating a hydroxyl group and at 1,705 cm^−1^ caused by a carbonyl function. The ESI-MS showed signals at *m/z* 185.1 [M+H]^+^ and 207.0 [M+Na]^+^ which was consistent with the calculated mass of *m/z* 184.1 for the proposed structure. The ^1^H spectrum did not show any aromatic proton signals. The ^13^C spectrum exhibited ten signals including a typical signal for a lactone functionality at 175.7 ppm. A ^13^C signal at 19.8 ppm with a corresponding ^1^H signal at 1.13 ppm for three protons suggested the presence of a methyl group (C-11). The methylene groups next to an oxygen gave characteristic ^13^C signals at 61.7 and 68.7 ppm (C-3 and C-10, respectively). The remaining signals were found in the aliphatic region. The ^1^H-^1^H COSY data showed the presence of a spin system involving the hydrogens H-5 to H-9 and, therefore, suggested the presence of a 5-membered ring. All signals have been fully assigned by means of ^1^H-^1^H COSY, HMBC and HMQC experiments. The configuration was determined by using coupling constants and NOESY data (*cf.* Figure 4). NOESY correlations between H-11 and H-9, H-5 and H-10, respectively, suggested the methyl group (C-11) and the proton at C-9 as well as the methylene group at C-10 and the proton at C-5 to be located in the same plane of the molecule. The small coupling constant of H-4 and H-3b (3.2 Hz) supports this hypothesis of a quasi-equatorial position of H-4. Additionally, the coupling constant between H-9 and H-8 (*J*_aa_ = 8.1 Hz) was backing a *trans*-configuration. Furthermore, the coupling constant between H-5 and H-9 (11.0 Hz) was consistent with the reported values of the structurally similar jatamanines having the same configuration [12]. Taking biogenetic reasons into account, compound **5** can be assigned to (4*R*,5*S*,8*R*,9*S*)-4-(hydroxymethyl)-8-methylhexahydro-cyclopenta[*c*]pyranone (villoside aglycone).

### 2.4. Valtrates with Antileishmanial Activity 

**Figure 5:**
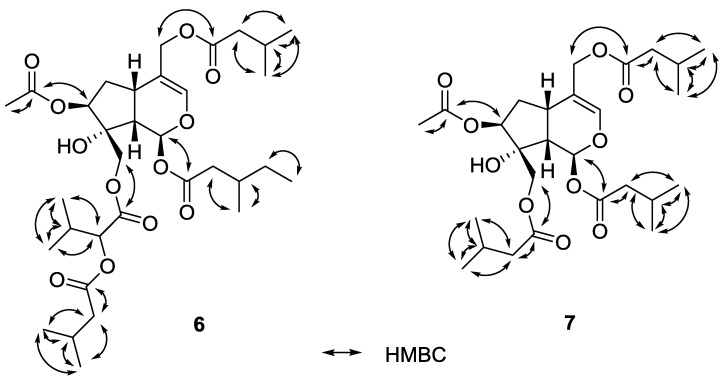
Key HMBC correlations of valtrates **6** and **7**.

Typical and well known constituents of *Valeriana* species are iridoid esters e.g., of the valepotriate and valtrate type, which have been previously investigated in detail because of their *in vitro* antifungal [14] and cytotoxic activity [15,16,17] and their potential use as antitumor agents [18]. In the active fractions F4–F7 two known valtrates were found, one (compound **8**) of the diene- and one (compound **9**) of the hydrine-type [16], and two iridoid esters **6**, **7** with an as yet unreported substitution pattern. The known valtrates were identified by comparing their spectral data to the literature data (*cf.* Table 1). Compound **8** has been reported before [14,19,20] and valechlorine (**9**) too [17,21,22]. The new compounds consist of a typical valtrate skeleton of the hydrine-type and are esterified with various acids e.g. isovaleric acid, acetic acid. The connections of the esters were assigned by HMBC experiments (*cf.* Figure 5). By comparing coupling constants and taking biogenetic considerations into account the configuration was determined to be the same as reported in the literature.

Most of the terpenes have been found in subfractions of F7 and F8 (*cf.* Figure 2). β-Bisabolol (**10**) [23], α-kessyl alcohol (**11**) [24,25], valeranone (**12**) [26] and bornyl isovalerate (**13**) are well known in the *Valeriana* species [27,28,29,30] since a plethora of terpenes has been identified from the essential oil of *Valeriana*, especially by GC/MS analysis [31,32] in the past. Linarin-*O*-2-methylbutyrate (**14**) has been reported from *V. wallichii* before [33]. Additionally the following known natural products were isolated from *V. wallichii* for the first time: the phenylpropanoids caffeic (**1**), isoferulic (**2**) and *p*-hydroxycinnamic acid bornyl ester (**3**), the lignans 4´-demethylpodophyllotoxin (**15**), podophyllotoxin (**16**) and pinoresinol (**17**). 

**Table 1:** ^1^H- and ^13^C-NMR data of new and known valtrates **6**–**9** in CDCl_3_.

	Position	^13^C of 6	^1^H of 6	^13^C of 7	^1^H of 7	^13^C of 8 [20]	^1^H of 8	^13^C of 9 [17]	^1^H of 9 [17]
	1	89.2	6.22 (d; 4.7; 1H)	89.2	6.27 (d; 4.2; 1H)	92.5	6.25 (d; 10.0; 1H)	89.4	6.19 (d; 5.1; 1H)
	3	141.0	6.44 (br s; 1H)	140.9	6.44 (br s; 1H)	148.0	6.68 (br s; 1H)	141.0	6.45 (br s; 1H)
	4	112.8		112.9		108.7		113.0	
	5	31.8	2.90 (dd; 9.2; 11.0; 1H)	31.4	2.90 (dd; 7.8; 8.0; 1H)	139.1		32.6	2.91 (m; 1H)
	6	34.9	2.1 (m; 2H)	34.7	2.12 (m; 2H)	117.6	5.77 (t; 2.8; 1H)	35.0	2.04–2.13 (m; 2H)
	7	80.1	4.99 (t; 4.5; 1H)	80.4	5.03 (t; 4.7; 1H)	83.1	5.47 (d; 2.8; 1H)	80.0	5.01 (t; 3.9; 1H)
	8	80.9		80.9		80.2		81.8	
	9	44.5	2.42 (dd; 6.1; 15.0; 1H)	44.7	2.44 (dd; 4.1; 9.6; 1H)	48.4	2.94 (dd; 2.5; 10.0; 1H)	45.4	2.45 (dd; 5.9; 8.1; 1H)
	10	66.8	4.29 (d; 11.5; 1H) 4.35 (d; 11.5; 1H)	66.6	4.22 (s; 2H)	65.5	4.32 (d; 11.6; 1H) 4.39 (d; 11.6; 1H)	49.1	3.71 (d; 11.4; 1H) 3.82 (d; 11.4; 1H)
	11	63.4	4.42 (d; 12.3; 1H) 4.62 (d; 12.3; 1H)	63.3	4.42 (d; 12.3; 1H) 4.62 (d; 12.3; 1H)	60.9	4.64 (d; 12.5; 1H) 4.71 (d; 12.5; 1H)	63.3	4.42 (d; 12.3; 1H) 4.60 (d; 12.3; 1H)
R^1^	1	171.3		171.1		170.8 ^d^		171.1	
	2	41.3	2.40 (m; 2H)	43.3 ^b^	2.23 (m; 4H)	43.2	2.32 (d; 7.0; 2H)	43.4 ^g^	2.09–2.13 (m; 4H)
	3	31.8	1.90 (m; 1H)	25.6 ^c^	2.07–2.15 (m; 3H)	25.7	2.05–2.09 (m; 2H)	25.7 ^h^	2.00–2.06 (m; 2H)
	4	19.5	0.97 (m; 3H)	22.3 ^a^	0.96 (m; 18H)	22.3 ^f^	0.93 (d; 6.6; 6H) ^g^	22.4 ^i^	0.97 (d; 6.7; 6H) ^g^
	5	29.3	1.40 (m; 2H)	22.3 ^a^	0.96 (m; 18H)	22.3 ^f^	0.93 (d; 6.6; 6H) ^g^	22.4 ^i^	0.97 (d; 6.7; 6H) ^g^
	6	11.2	0.90 (m; 3H)						
R^2^	1	169.7		173.0		170.8 ^d^		172.9	
	2	43.0	2.30–2.35 (m; 4H)	43.5	2.19 (m; 2H)	20.9	2.04 (s; 3H)	43.3 ^g^	2.09–2.13 (m; 4H)
	3	25.7	2.11 (m; 2H)	25.7 ^c^	2.07–2.15 (m; 3H)			25.6 ^h^	2.00–2.06 (m; 2H)
	4 and 5	22.4	0.98 (d; 6.9; 12H)	22.4 ^a^	0.96 (m; 18H)			22.4 ^i^	0.95 (d; 6.6; 6H) ^g^
R^3^	1	170.2		170.3		171.8		169.8	
	2	21.0	2.07 (s; 3H)	21.0	2.05 (s; 3H)	43.4	2.14 (m; 2H)	21.0	2.08 (s; 3H)
	3					25.6 ^e^	2.05–2.09 (m; 2H)		
	4 and 5					22.3 ^f^	0.95 (d; 6.6; 6H) ^g^		
R^4^	1	173.2		172.9		173.8			
	2	76.9	4.74 (d; 4.8; 1H)	43.1 ^b^	2.23 (m; 4H)	43.0	2.20 (m; 2H)		
	3	30.0	2.23 (m; 1H)	25.7 ^c^	2.07–2.15 (m; 3H)	25.6 ^e^	2.17–2.25 (m; 1H)		
	4	18.8	1.02 (m; 3H)	22.3 ^a^	0.96 (m; 18H)	22.3 ^f^	0.99 (d; 6.6; 6H) ^g^		
	5	17.3	1.00 (m; 3H)	22.3 ^a^	0.96 (m; 18H)	22.3 ^f^	0.99 (d; 6.6; 6H) ^g^		
	6	172.9							
	7	43.0	2.30–2.35 (m; 4H)						
	8	25.7	2.11 (m; 2H)						
	9 and 10	22.4	0.98 (d; 6.9; 12H)						

^a–i^ Assignments may be interchanged.

### 2.5. Antileishmanial Activity and Cytotoxicity 

All compounds isolated were subjected to the evaluation of their antileishmanial activity against *L. major* promastigotes *in vitro* and cytotoxicity against macrophages (J774.1 murine cell line) using the corresponding AlamarBlue^©^ assays [34] (*cf.* Table 2). 

**Table 2:** Antileishmanial activity and cytotoxicity of fractions containing isolated compounds.

Compound	IC_50_ (*L. major*) [µg/mL]	IC_50_ (J774.1) [µg/mL]
**Bornyl caffeate (1)**	48.8 *	8.3 *
**2**	16.7	34.7
**3**	12.2	8.6
**4**	>100	>100
**5**	>100	17.5
**6**	**1.9 ^a^**	**<0.8 ^a^**
**7**	**0.8**	**<0.8**
**8**	1.7 *	1.0 *
**9**	**2.3**	**1.9**
**β-Bisabolol (10)**	52.2	37.1
**α-Kessyl alcohol (11)**	**5.8** ^b^	**1.7** ^b^
**Valeranone (12)**	60.8	42.6
**Bornyl isovalerate (13)**	>100 *	>100 *
**Linarin-*O*-2-methylbutyrate (14)**	>100	10.8
**15**	>100	<**0.8**
**16**	>100	**<0.8**
**Pinoresinol (17)**	>100	<**0.8**
**Miltefosine**	36.2 *	56.5 *

* These data were gained from pure or synthesized compounds and are therefore given in µM. ^a^ There was a small amount of another valtrate present. ^b^ The fraction of kessyl alcohol was contaminated with an non-identified valtrate (~10% by NMR integration).

The valtrates **6**–**9** showed the highest antileishmanial activity (IC_50_ 0.8–2.3 µg/mL). Their cytotoxicity is in the same range. It is unknown whether the cytotoxicity is directly responsible for the leishmanicidal potential. Nevertheless, the valtrates represent the main active principle of the extract. Fractions with moderate antileishmanial activity in the range of the standard drug miltefosine were phenylpropanoids bornyl caffeate (**1**), bornyl isoferulate (**2**) and bornyl 3-hydroxycinnamate (**3**). This has led to the synthesis of a compound library and QSAR studies recently reported by us [2]. 

Furthermore, valeranone (**12**) and β-bisabolol (**10**) exhibited moderate antileishmanial and cytotoxic activity. The kessyl alcohol fraction showed rather good antileishmanial activity. However, it was contaminated with an active but unidentified valtrate, thus it remains unclear whether kessyl alcohol (**8**) is really active. Bornyl isovalerate (**13**), linarin-*O*-2-methylbutyrate (**14**), podophyllotoxin (**11**), 4'-demethylpodophyllotoxin (**15**) and pinoresinol (**16**) showed no antileishmanial activity. However, podophyllotoxin and 4'-demethylpodophyllotoxin were highly cytotoxic (IC_50_ < 0.8 µg/mL). This is not surprising as podophyllotoxin and derivatives are widely used as anticancer agents. However, cytotoxicity and antileishmanial activity are not linked to each other in this case. Fractions with novel compounds **4** and **5** exhibited no antileishmanial activity (IC_50_ > 100 µg/mL). Nevertheless, villoside aglycone (**5**) showed moderate cytotoxicity (IC_50_ 17.5 µg/mL). Taken together the antileishmanial activity was found in valtrate derivatives, mainly, and cinnamic acid derivatives with exception of novel compound **4**. However the valtrates were found to be highly cytotoxic in contrast to the cinnamic acid derivatives having moderate cytotoxicity.

## 3. Experimental Section 

### 3.1. General

^1^H (400.132 MHz), ^13^C (100.613 MHz) and 2D NMR spectra were recorded on a Bruker Avance 400 Ultra Shield™ (Bruker Biospin, Ettlingen, Germany) spectrometer. As internal standard the signals of the deuterated solvents were used (CDCl_3_: ^1^H 7.26 ppm, ^13^C 77.0 ppm). IR spectra were recorded on a Jasco FT/IR-6100 spectrometer (Groß-Umstadt, Germany) with an ATR unit at room temperature. LC/MS was conducted on an Agilent 1100 analytical HPLC with DAD detection and an Agilent LC/MSD Trap (Agilent Technologies, Böblingen, Germany). For flash chromatography a Reveleris Flash system (Grace, Columbia, MD, USA) was used. Preparative HPLC was conducted using an Agilent 1100 preparative HPLC with fraction collector and multiple wavelength detector (MWD). TLC plates (60 F254) were purchased from Macherey-Nagel. Column chromatography was carried out on silica gel (Kieselgel 60, 0.063–0.2 mm, 70–320 mesh, Merck, Darmstadt, Germany), filtration on LiChroprep© RP-18 (40–63 µm, Merck).

### 3.2. Plant Material

Dried rhizomes of *V. wallichii* were acquired from a local herb shop in Kolkata, India. The identity as *V. jatamansi* Jones syn. *V. wallichii* DC. was confirmed by comparison with an authentic sample cultivated at the Institute of Himalayan Bioresource Technology in Palampur, Himachal Pradesh, India by R.D. Singh. A voucher specimen of the herbarium was preserved in the laboratory of Banasri Hazra (IHBT Ref. No. 11666).

### 3.3. Extraction and Isolation

The pulverized rhizome (10 g) was refluxed with chloroform (100 mL) for 2 h and filtered. The solvent was removed from the filtrate in a rotary evaporator, followed by complete drying *in vacuo*. Thus, the chloroform extract was obtained (yield = 1.1%; *w/w*) and preserved at 4 °C. The process was repeated to get higher amounts of the extract as per experimental requirements. Dry extract (11 g) was slurried in MeOH and filtrated using a short column filled with LiChroprep RP-18 silica gel which yielded 9.5 g of extract after evaporation. This material was fractionated into 12 fractions (F1–F12) by *preparative HPLC method A* (250 mm × 10 mm, 5 µm, Macherey-Nagel Nucleosil 100-5; A) H_2_O, B) MeOH, gradient: 70% B (0 min), 75% B (7 min), 100% B (25 min), 70% B (30 min); flow rate: 3.3 mL/min). 

A portion of 600 mg of fraction F1 was further fractionated by *preparative HPLC method B* (125 mm × 10 mm, 5 µm, Macherey-Nagel Nucleodur Sphinx RP; A) H_2_O, B) CH_3_CN, gradient: 10% B (0–2 min), 30% B (2–15 min), 65% B (15–22 min), 10% B (22–24 min); flow rate: 4.4 mL/min) resulting in 9 fractions (F1-1 to F1-9). F1-1 (1.5 mg) yielded villosid aglycone **5**, F1-4 (9 mg) the novel nepetolactone derivative **4**. From F1-5 (3.7 mg) and F1-6 (9.1 mg) podophyllotoxin (**16**) and 4'-demethylpodophyllotoxin (**15**) were identified and F1-7 (2.9 mg) contained pinoresinol (**17**). 

A 150 mg portion of fraction F2 was partitioned in two fractions (F2-I and F2-II) using the above *HPLC method B*. In F2-I linarin-*O*-2-methylbutyrate (**14**) (6.0 mg) was found and F2-II (12.5 mg) yielded podophyllotoxin (**16**) 

Fractionation of F3 into three fractions (F3-I to F3-III) yielded again the linarin derivative **14** in F3-II. Fraction F4 was subjected to flash chromatography (silica gel, MeOH/CHCl_3_, 4.8:0.2 *v/v*) and gave 6 fractions (4α–4ζ) from which valechlorine (**9**) was identified in fraction 4γ (32.6 mg).

Fraction F5 was separated by column chromatography (CC) on silica gel (MeOH/CHCl_3_, 4.8:0.2 v/v) and yielded 5 fractions (5α, 5β1, 5β2, 5β3, 5γ). Repeating the chromatography with fraction 5β3 (258 mg) gave three fractions (5β3a–5β3c) of which the last fraction 5β3c yielded 135 mg of bornyl caffeate (**1**). Fractionation of 5β2 (168 mg) by CC gave four fractions (5β2a–5β2c) of which fraction 5β2a gave 14.2 mg of valtrate **7**. Fractions 5β2b and 5β2c were combined and subjected to *preparative HPLC method B*). This process resulted in six fractions (5β2bc1–5β2bc6) from which fraction 5β2bc yielded 6.1 mg of valtrate **9**. 

Fraction F6 was again fractionated like F5 by CC which gave seven fractions 6α–6η. 6γ (24.2 mg) and 6δ (25.7 mg) were combined and subjected to *preparative HPLC method B* which gave fractions 6dega_1_-6dega_5_. 6dega_2_ again yielded valtrate **7** (4.9 mg) and 6dega_3_ valtrate **8** (4.0 mg). 

F7 was fractionated by column chromatography as above and gave four fractions (7α–7δ). 7α (57.6 mg) was further partitioned by CC into four fractions (7α_1_–7α_4_) of which 7α_2_ yielded 1.5 mg of a mixture of valeranone (**12**) and β-bisabolol (**10**). Further fractionation of 7α_3_ (19.7 mg) by the same CC method gave another 3 fractions (7α_3a_–7α_3c_) of which 7α_3a_ consisted of 5.0 mg pure β-bisabolol (**10**). 7α_3c_ yielded 2.1 mg of caffeic acid derivative **2**. Fractions 7β and 7γ were combined and fractionated by *preparative HPLC method B* which resulted in 10 fractions (7bega_1_–7bega_10_) of which fraction 7bega_2_ gave 7.6 mg cinnamic acid derivative **3** and fraction 7bega_9_ 2.5 mg of valtrate **6**. 

F8 was fractionated into 6 fractions (8α–8ζ) by CC. Fraction 8β yielded 1.7 mg of bornyl isovalerate (**13**). 8ε was fractionated further by *HPLC method B* and gave six fractions (8ε_1_–8ε_6_) with fraction 8ε_4_ yielding 6.8 mg kessyl alcohol (**11**). 

### 3.4. Characterization of Novel Nepetolactone Derivative {[(3S,4S,7R,8S)-1-Oxo-3,7-dimethyl-4-(O-β-d-glucopyranosyl)oxy]hexahydrocyclopenta[c]furan-3-yl}-methyl-trans-4-hydroxycinnamate *(**4**)*

Yellowish syrup; IR (ATR): 3382 (OH); 1749, 1697 (C=O), 1603 (C=C) cm^-1^; ^1^H-NMR (CDCl_3_): δ = 1.19 (3H, d, *J* = 6.9 Hz, H-11), 1.73 (3H, s, H-9), 1.41 (1H, m, H-6b), 1.68 (1H, m, H-5b), 1.97 (1H, m, H-6a), 2.20 (1H, m, H-5a), 2.35 (1H, m, H-7), 2.83 (1H, m, H-8), 3.22 (1H, m, H-2'), 3.26 (1H, m, H-5'), 3.41 (1H, t, *J* = 9.1 Hz, H-3'), 3.50 (1H, t, *J* = 9.6 Hz, H-4'), 3.79 (2H, dd, *J* = 3.2, 7.6 Hz, H-6'), 4.37 (1H, d, *J* = 7.5 Hz, H-1'), 4.52 (2H, m, H-10), 6.21 (1H, d, *J* = 15.9 Hz, H-2"), 6.79 (2H, *J* = 8.6 Hz, H-6", H-8"), 7.36 (2H, d, *J* = 8.6 Hz, H-5", H-9"), 7.60 (1H, d, *J* = 15.9 Hz, H-3") ppm; ^13^C-NMR (CDCl_3_): δ = 17.1 (C-9), 21.5 (C-11), 30.6 (C-5), 33.0 (C-6), 37.6 (C-7), 59.8 (C-8), 61.3 (C-6'), 67.3 (C-10), 69.4 (C-4'), 73.2 (C-2'), 76.0 (C-5'), 76.3 (C-3'), 86.0 (C-4), 93.4 (C-3), 96.8 (C-1'), 113.5 (C-2"), 115.8 (C-6", C-8"), 125.7 (C-4"), 130.2 (C-5", C-9"), 146.4 (C-3"), 159.5 (C-7"), 167.5 (C-1"), 177.2 (C-1) ppm; ESI-MS: *m/z* 621.4 [M−H+TFA]^−^, 506.9 [M−H]^−^, 326.8 [M−Glc]^−^; HRMS (ESI-TOF^+^): *m/z* [M+Na]^+^ calculated for C_25_H_32_O_11_Na: 531.1842; found: 531.1836.

### 3.5. Characterization of Villoside Aglycone (4R,5S,8R,9S)-4-(Hydroxymethyl)-8-methylhexahydro-cyclopenta[c]pyranone *(**5**)*

Yellowish syrup; IR (ATR): 3387 (OH); 1705 (C=O) cm^−1^; ^1^H-NMR (CDCl_3_): δ = 1.11–1.18 (1H, m, H-7a), 1.13 (3H, d, *J* = 6.4 Hz, H-11), 1.21–1.31 (1H, m, H-6a), 1.63–1.72 (1H, m; H-4), 1.82–1.88 (1H, m, H-7b), 1.97–2.05 (1H, m, H-6b), 2.12–2.27 (2H, m, H-8, H-5), 2.32 (1H, dd, *J* = 8.4, 11.0 Hz, H-9), 3.44 (1H, dd, *J* = 8.00, 11.1 Hz, H-10a), 3.64 (1H, dd, *J* = 4.70, 11.1 Hz, H-10b), 4.10 (1H, m, H-3a), 4.35 (1H, dd, *J* = 3.20, 11.1 Hz, H-3b) ppm; ^13^C-NMR (CDCl_3_): δ = 19.8 (C-11), 32.3 (C-6), 34.6 (C-7), 38.4 (C-8), 38.9 (C-5), 42.4 (C-4), 48.9 (C-9), 61.7 (C-10), 68.7 (C-3), 175.7 (C-1) ppm; ESI-MS: *m/z* 185.1 [M+H]^+^, 207.0 [M+Na]^+^; HRMS (ESI-TOF^+^): *m/z* [M+Na]^+^ calculated for C_10_H_16_O_3_Na: 207.0997; found: 207.0993.

### 3.6. Antileishmanial and Cytotoxicity Assays

Antileishmanial activity against *L. major* promastigotes and cytotoxicity against a murine macrophage cell line (J774.1) was determined using an AlamarBlue^©^ assay. Methods for both procedures have been reported previously [34]. IC_50_ values are presented as mean values of two experiments.

## 4. Conclusions 

The valtrates **6**–**9** represent the main active antileishmanial principles of the *V. wallichii* extract, but they are toxic. Additionally, the cinnamic acid derivatives **1**–**3** showed moderate activity, interestingly except for novel compound **4**. This could be due to the larger size of the molecule or the higher polarity caused by the sugar moiety. However, the previously reported cinnamic acid derivatives exhibited higher activity, even without the double bond [2].
